# Employment status and diabetic outpatient appointment non-attendance in middle to senior working generation with type 2 diabetes: the Japan diabetes outcome intervention trial-2 large‑scale trial 005 (J-DOIT2-LT005)

**DOI:** 10.1007/s00592-022-01869-0

**Published:** 2022-03-12

**Authors:** Izumi Nakayama, Atsushi Goto, Yasuaki Hayashino, Hikari Suzuki, Katsuya Yamazaki, Kazuo Izumi, Mitsuhiko Noda

**Affiliations:** 1 Department of Health Data Science, Graduate School of Data Science, Yokohama City University, 22-2, Seto, Kanazawa-ku, Yokohama, Kanagawa 236-0027 Japan; 2 Department of Endocrinology, Tenri Hospital, 200 Mishimacho, Nara, Tenri Japan; 3 Japan Community Health Care Organization Takaoka Fushiki Hospital, 8-5 Fushiki Kofumotomachi, Takaoka, Toyama Japan; 4 Kawai Clinic, 715-1 Higashihiratsuka, Tsukuba, Ibaraki Japan; 5 Center for Clinical Sciences, National Center for Global Health and Medicine, 1-21-1 Toyama, Shinjuku-ku, Tokyo, Japan; 6 Department of Diabetes, Metabolism and Endocrinology, Ichikawa Hospital, International University of Health and Welfare, Ichikawa, Chiba Japan

**Keywords:** Employment status, Non-attendance, Type 2 diabetes mellitus, Primary care

## Abstract

**Aims:**

Workplace demands, support, and relationships differ according to employment status (e.g., employment that is full-time, part-time, or self-employed) and may lead to unequal opportunities to keep diabetic appointments. We investigated the association between employment status and outpatient diabetic appointment non-attendance among working-age adults with type 2 diabetes.

**Methods:**

This was a secondary analysis of a cluster-randomized trial (the Japan diabetes outcome intervention trial 2 large-scale trial). The analysis included 2010 trial participants (40–65 years old) with type 2 diabetes who were regularly followed by primary care physicians (PCPs). The outcome measure was the first non-attendance (defined as a failure to visit a PCP within 2 months of the original appointment) during the one-year follow-up. The association between baseline employment status and non-attendance was examined using Cox proportional hazard model in men and women.

**Results:**

During the 1279 and 789 person-year follow-up periods, 90 men and 34 women, respectively, experienced their first appointment non-attendance. Among men, self-employed participants had a higher risk of non-attendance compared with full-time employees (adjusted HR, 1.84; 95% CI, 1.15, 2.95). The trial intervention (attendance promotion) was associated with a significantly reduced risk of non-attendance among self-employed participants (HR, 0.51; 95% CI, 0.26, 0.99). Among women, a significant association between employment status and non-attendance was not observed.

**Conclusions:**

Self-employed men with type 2 diabetes had a twofold increased risk of non-attendance than did full-time employees. Our study suggests that self-employed men with type 2 diabetes should be targeted for interventions promoting appointment adherence.

**Supplementary Information:**

The online version contains supplementary material available at 10.1007/s00592-022-01869-0.

## Introduction

Inadequate control of type 2 diabetes mellitus leads to increased cardiovascular complications and premature death. Non-attendance at regular diabetes outpatient appointments interrupts the continuity of care and impairs the quality of diabetes management. Among people with type 2 diabetes, non-attendance at these appointments is common and is associated with suboptimal glycemic control [1].

Three of four people with diabetes are working age (20–64 years old), and the prevalence of diabetes in this age-group is expected to increase [2]. Preventing diabetes progression in the working-age population has a substantial impact on individual health and population-level work productivity [3]. Increasing evidence suggests that work-related factors, such as workplace demands, support, and relationships, affect health behaviors [4]. These factors differ according to employment status (e.g., employment that is full-time, part-time, or self-employed) and may lead to unequal opportunities to keep regular medical appointments [5]. However, there is insufficient evidence to confirm the existence of an association between employment status and health appointment non-attendance [1, 6].

Recently, a cluster-randomized trial among adults (40–65 years old) with type 2 diabetes showed that a multifaceted intervention program reduced the risk of non-attendance at regular primary care appointments [7]. The intervention consisted of reminders, patient lifestyle modification education, and clinical performance feedback for primary care providers. If deployed effectively, the study suggested that such programs may contribute to workers maintaining their continuity of diabetes care.

This study investigated the association between employment status and non-attendance at diabetic outpatient appointments among working-age adults with type 2 diabetes. Additionally, in employment status with an increased risk for appointment non-attendance, the study examined the effect of appointment attendance promotion intervention of Japan Diabetes Outcome Intervention Trial 2 large-scale trial (J-DOIT2-LT) [7]. Our goal was to better understand working-age people who have an increased risk for appointment non-attendance and, thereby, facilitate the effective allocation of interventions that promote adherence to diabetes outpatient appointments.

## Methods

### Study population

The study population included participants in J-DOIT2-LT, a cluster-randomized trial that evaluated the effect of a three-faceted approach that aimed to promote attendance at regular primary care appointments among adult participants with type 2 diabetes [7]; the trial protocol is available elsewhere [8]. Briefly, the trial involved 11 district medical associations (DMAs) across a broad area of Japan and involved 192 primary care physicians (PCPs). In each DMA, the PCPs were divided into two geographic clusters. Thereafter, the 22 clusters were randomly assigned to either the intervention or control group, stratified by DMA. Eligible participants were enrolled between July and September 2009. Participants were eligible to participate if they were 40–65 years old and had an established type 2 diabetes diagnosis. Participants were excluded if they had type 1 diabetes mellitus; had a history of lower limb amputation or a malignant tumor within the preceding 5 years; or were on hemodialysis, pregnant, hospitalized, residing in a nursing home, or bed-ridden. For the present analysis, we further excluded participants who were retired or had missing baseline employment status information.

In the intervention group, participants received mail or telephone reminders to attend their regular appointments and six lifestyle modification education sessions conducted by trained specialists. In addition, the PCPs received monthly quality indicator feedback regarding the diabetes care they provided. The interventions continued for one year. In the control group, standard diabetes care, based on the latest practice guidelines, was provided.

### Exposures and covariates

Variables used in the analysis were obtained in the following manner. At baseline, the clinical research coordinators (CRCs) reviewed each participant’s medical records and collected baseline characteristics, including age, sex, body mass index (BMI), blood pressure, glycated hemoglobin (HbA1c) measurements, and medications. Upon enrollment, participants completed a self-administered questionnaire that asked about their smoking history, history of non-attendance at medical appointments, current employment status, average weekly working hours, and the number of years that had elapsed since registering with their PCP.

The main exposure variable was baseline employment status. In a self-administered questionnaire, participants were asked to choose the most appropriate category to describe their current employment status: ‘full-time employee,’ ‘part-time employee,’ ‘full-time homemaker,’ ‘retired,’ ‘never had a regular job,’ ‘disabled and unemployed,’ ‘self-employed,’ or ‘unemployed.’ Regarding employment, a small proportion of participants (1.7%) chose more than one category. These participants were assigned to the most appropriate category, based on the judgment of the trial management team. We collapsed the categories of ‘never had a regular job,’ ‘disabled and unemployed,’ and ‘unemployed’ into a single category called ‘unemployed.’ This was done because of the small numbers of participants who had never had a regular job (N = 2) or were disabled and unemployed (N = 15). Accordingly, we considered employment status as a categorical exposure variable with five levels: full-time employee, part-time employee, self-employed, unemployed, and full-time homemaker.

### Outcome measure

The outcome measure was the first missed appointment (non-attendance) defined as a failure to visit a PCP within 2 months of the original appointment. In Japan, PCPs adjust the interval to the next appointment according to their patients’ condition at each appointment. The day of the event was defined as two months plus one day from the missed appointment (the planned next appointment). The follow-up began at the time of randomization and ended at the first non-attendance, when the patient was lost to follow-up, or at the end of the study period (October 2010), whichever occurred first. Trial CRCs ascertained the reason for non-attendance based on participant medical records. Non-attendances due to explicit causes (e.g., referral to another clinic, hospital admission, or moving) were excluded.

### Statistical analysis

We analyzed men and women separately because the association with employment status was assumed to be qualitatively different between the sexes. Baseline characteristics were analyzed using the Kruskal–Wallis or Fisher’s exact tests. HbA1c measurements were originally collected in the Japan Diabetes Society (JDS) units (%). The HbA1c values were reported in the International Federation of Clinical Chemistry and Laboratory Medicine (IFCC) units (mmol/mol) and National Glycohemoglobin Standardization Program (NGSP) units (%) following appropriate conversions (NGSP [%] = 1.02*JDS [%] + 0.25, IFCC [mmol/mol] = 10.93*NGSP [%]–23.50) [9]. The crude incidence of non-attendance per 1000 person-years and the associated 95% CIs were calculated assuming a Poisson distribution. We modeled the association between employment status and time to first non-attendance using a Cox proportional hazard regression analysis to estimate the HRs and 95% CIs. We used robust variance estimation to account for correlations within clusters (DMAs) and within assigned treatment groups. In Model 1, we adjusted for age. In Model 2, we further adjusted for HbA1c level, receiving diabetes treatment, BMI, and history of previous non-attendance. The selection of potential confounding factors was based on a directed acyclic graph organized with previous knowledge about the exposure and outcome predictors (Supplementary figure). Additionally, because we found that self-employed men had a higher risk of non-attendance, we evaluated the effect of appointment adherence promotion on non-attendance in self-employed men and employment status. We estimated the unadjusted HR for the intervention on appointment non-attendance using a Cox proportional hazard regression model and constructed Kaplan–Meier curves for time to non-attendance among self-employed men. In each regression analysis, we assumed that missing variables were missing at random and imputed them with multivariate imputation using chained equations. We included the patient characteristics shown in Table 1, medications, cluster identification, employment status, time to event, and events in the imputation model. We fitted the models to 100 copies of the imputed data and pooled the estimates using Rubin’s principle [10]. In addition, we compared the results with those of complete case analyses. The analyses were performed using R (version 4.0.3 and the mice package version 3.13.0, R Foundation for Statistical Computing, Vienna, Austria). This study was approved by the Yokohama City University institutional review board. Because this secondary analysis used existing, de-identified trial data, the requirement for informed consent was waived.

**Table 1:** Participant baseline characteristics by employment status in men and women

Men						
		Full-time employee N = 819 (66%)	Part-time employee N = 52 (4%)	Unemployed N = 100 (8%)	Self-employed N = 278 (22%)	*p*-value
Age, year		56 (50, 59)	61 (59, 63)	60 (57, 62)	59 (54,61)	< 0.001
BMI, kg/m^2^		25.6 (23.3, 28.4)	24.3 (21.6, 26.6)	24.9 (22.9, 27.9)	25.1 (23.0, 27.5)	0.020
Missing		147	7	17	50	
Current smoker, N (%)		320 (42%)	28 (58%)	42 (46%)	108 (42%)	0.131
Missing		49	4	9	23	
Antihypertensive therapy, N (%)		443 (54%)	34 (65%)	52 (52%)	158 (57%)	0.353
Lipid-lowering therapy, N (%)		352 (43%)	17 (33%)	38 (38%)	88 (32%)	0.006
ACE or ARB, N (%)		321 (39%)	25 (48%)	39 (39%)	118 (42%)	0.504
HbA1c, mmol/mol		54 (47, 62)	55 (48, 63)	53 (47, 64)	54 (47, 63)	0.910
HbA1c, %		7.08 (6.47, 7.80)	7.19 (6.57, 7.90)	6.98 (6.47, 8.00)	7.08 (6.47, 7.90)	0.910
Missing		42	3	3	5	
Treatment for diabetes, N (%)						0.455
No medication		66 (8%)	2 (4%)	6 (6%)	22 (8%)	
Oral agents		677 (85%)	41 (84%)	80 (82%)	220 (82%)	
Insulin		57 (7%)	6 (12%)	11 (11%)	26 (9%)	
Missing		19	3	3	10	
Weekly working hours		40 (10, 50)	24 (12, 37)	0 (0, 7)	32 (10, 50)	< 0.001
Missing		27	1	76	4	
Years followed by the PCP		4 (2, 9)	5 (2, 13)	5 (2, 8)	5 (2, 10)	0.182
Missing		54	3	5	18	
History of non-attendance, N (%)						0.200
None		673 (83%)	46 (90%)	76 (78%)	224 (82%)	
Once		90 (11%)	3 (6%)	11 (11%)	25 (9%)	
Twice or more		45 (6%)	2 (4%)	11 (11%)	23 (9%)	
Missing		11	1	2	6	

Women							
		Full-time employee N = 142 (19%)	Part-time employee N = 232 (30%)	Unemployed N = 81 (11%)	Self-employed N = 84 (11%)	Homemaker N = 222 (29%)	*P* value
Age, year		56.0 (52.0, 59.0)	58.0 (53.0, 61.0)	60.0 (57.0, 62.0)	60.0 (55.0, 62.0)	60.0 (56.0, 62.0)	< 0.001
BMI, kg/m^2^		25.9 (23.6, 28.8)	25.6 (22.6, 28.2)	25.7 (22.3, 29.2)	24.7 (23.1, 27.6)	25.9 (23.4, 29.5)	0.415
Missing		27	48	15	18	49	
Current smoking, N (%)		19 (15%)	29 (15%)	13 (18%)	14 (19%)	21 (11%)	0.456
Missing		19	35	9	10	37	
Antihypertensive therapy, N (%)		73 (51%)	132 (57%)	50 (62%)	44 (52%)	124 (56%)	0.601
Lipid-lowering therapy, N (%)		60 (42%)	117 (50%)	40 (49%)	38 (45%)	117 (53%)	0.346
ACE or ARB, N (%)		47 (33%)	82 (35%)	24 (30%)	26 (31%)	81 (36%)	0.772
HbA1c, mmol/mol		55 (47, 66)	54 (48, 63)	52 (46, 65)	53 (46, 62)	53 (48, 61)	0.471
HbA1c, %		7.19 (6.47, 8.21)	7.08 (6.57, 7.90)	6.88 (6.37, 8.10)	6.98 (6.37, 7.80)	6.98 (6.57,7.70)	0.471
Missing		9	12	3	5	2	
Treatment for diabetes, N (%)							0.831
No medication		10 (7%)	23 (10%)	8 (10%)	8 (9%)	16 (7%)	
Oral agents		115 (82%)	188 (83%)	65 (81%)	71 (85%)	178 (82%)	
Insulin		15 (11%)	16 (7%)	7 (9%)	5 (6%)	22 (10%)	
Missing		2	5	1	0	6	
Weekly working hours		40 (8, 44)	20 (8, 30)	4 (0, 16)	30 (8, 48)	14 (5, 30)	< 0.001
Missing		4	8	70	2	161	
Years followed by the PCP		4 (2, 8)	5 (2, 10)	5 (2, 9)	5 (2, 13)	4 (2, 10)	0.136
Missing		17	19	4	10	15	
History of non-attendance, N (%)							0.098
None		117 (83%)	197 (88%)	72 (90%)	70 (86%)	200 (93%)	
Once		17 (12%)	15 (7%)	8 (10%)	5 (6%)	10 (5%)	
Twice or more		7 (5%)	13 (6%)	0 (0%)	6 (7%)	6 (3%)	
Missing		1	7	1	3	6	

Data are presented as medians (interquartile range) or N (%) and analyzed using the Kruskal–Wallis or Fisher’s exact test. The number of missing values for each variable is shown. ACE, angiotensin -converting enzyme; ARB, angiotensin II receptor blockers; HbA1c, glycated hemoglobin; PCP, primary care physician

## Results

**Fig. 1:**
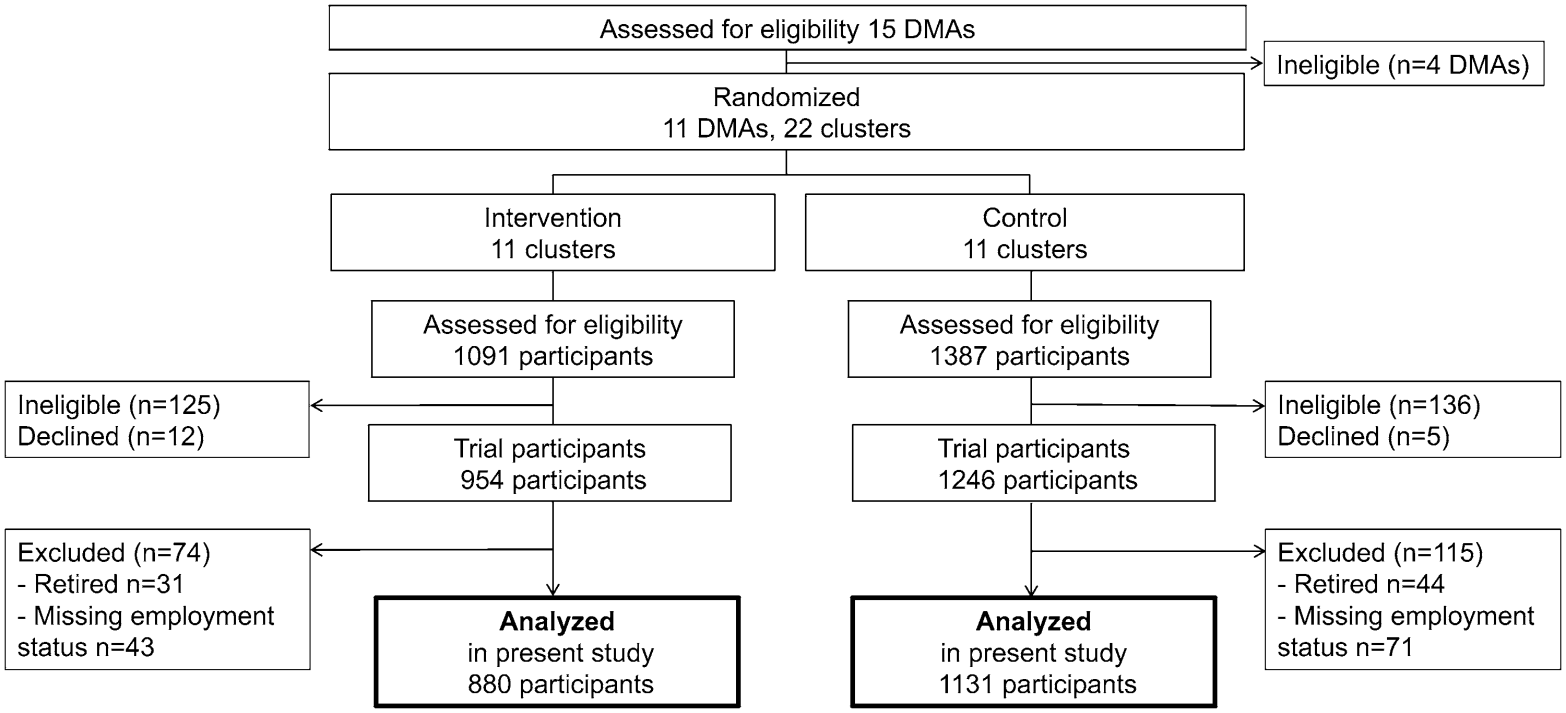
Study diagram of participants included in the present analysis from the Japan Diabetes Outcome Intervention Trial 2 large-scale trial (J-DOIT2-LT) DMAs, district medical associations

Among the 2200 participants in the J-DOIT2-LT, 2011 participants met the inclusion criteria and were included in this secondary analysis; 75 participants were excluded due to being retired, and 114 were excluded due to missing employment status records (Fig. 1). One male patient was also excluded due to being described as a full-time homemaker; therefore, the study included 1249 men (62.1%) and 761 women (37.9%). The median patient age was 58 (IQR, 53–61) years, and the median HbA1c level was 7.1% (IQR, 6.5–7.9) or 54 mmol/mol (IQR, 47–63); 1639 (81.5%) participants were taking oral diabetic medications and 165 (8.2%) were using insulin. The participants were classified as full-time employees (47.8%), part-time employees (14.1%), self-employed (18.0%), unemployed (9.0%), or full-time homemakers (11.1%). The employment status distribution differed between men and women (Table 1); the majority (66%) of the men were full-time employees, whereas, among women, the most common employment status was part-time employment (30%). For both sexes, full-time employees were younger than those in the other classifications at baseline (Table 1). The baseline distribution of HbA1c levels, participants receiving diabetes treatment, and history of non-attendance at appointments were similar across the various employment statuses. The numbers of missing data for each variable are also reported in Table 1. The proportions of missing variables in Model 2 were: BMI, 18.8% (378/2010); HbA1c level, 4.2% (84/2010); treatment for diabetes, 2.4% (49/2010); and history of appointment non-attendance, 1.9% (38/2010).

The median follow-up was 391 days in men and 392 days in women. For 90 men and 34 women, the first appointment non-attendance event occurred after 1279 and 789 person-years of follow-up, respectively. None of the participants were lost to follow-up, and outcome measures were ascertained in all participants. The crude incidence rate was higher for men than for women and was higher in the control group than in the intervention group (Supplementary Table 1).

The HRs for appointment non-attendance are presented in Table 2. The HRs for Model 1, Model 2 with complete case analysis, and Model 2 with multiple imputed data are compared in Supplementary Table 2. The complete case analyses for Model 2 included 77.0% (962/1249) of the men and 73.3% (558/761) of the women.

**Table 2:** Crude incidence rates and adjusted hazard ratios for non-attendance

Employment status	Person- Years	Non- attendance (N)	Rate/1000 person-years (95% CI)	Hazard ratio^a^ (95% CI)
*Men*				
Full-time	844.2	51	60.4 (45.0, 79.4)	1.0
Self-employed	278.6	30	107.7 (72.6, 153.7)	1.84 (1.15, 2.95)
Part-time	52.7	3	57.0 (11.7, 166.5)	1.10 (0.45, 2.66)
Unemployed	103.1	6	58.2 (21.4, 126.7)	1.02 (0.53, 1.93)
Homemaker	–	–	–	–
*Women*				
Full-time	146.6	8	54.6 (23.6, 107.5)	1.0
Self-employed	85.6	2	23.4 (2.8, 84.4)	0.44 (0.10, 1.98)
Part-time	237.3	16	67.4 (38.5, 109.5)	1.29 (0.52, 3.18)
Unemployed	83.7	3	35.8 (7.4, 104.7)	0.81 (0.20, 3.28)
Homemaker	235.5	5	21.1 (6.9, 49.5)	0.51 (0.13, 1.99)

Crude incidence rates were estimated assuming a Poisson distribution. Adjusted hazard ratios for the first appointment non-attendance are pooled estimates from a Cox proportional hazard regression model using robust variance (Model 2) fitted to multiply imputed data

^a^Adjusted according to baseline age, glycated hemoglobin level, treatment for diabetes, body mass index, and history of previous appointment non-attendance

**Fig. 2:**
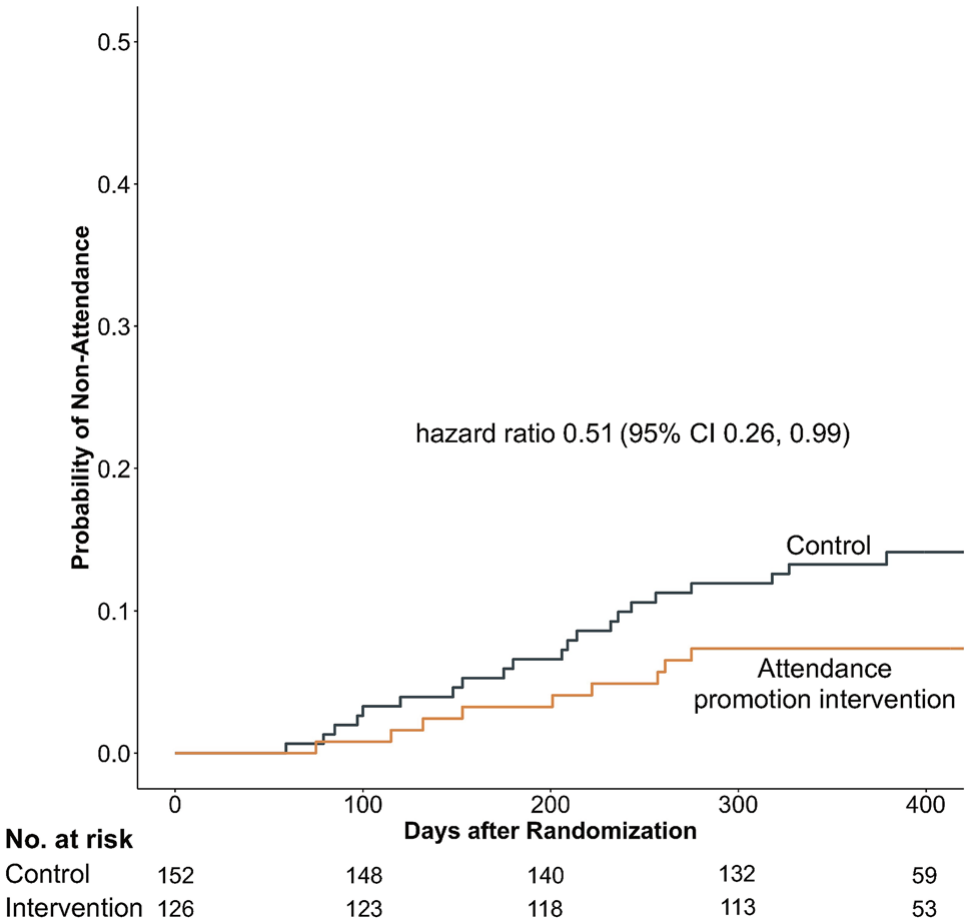
Survival curves for the first diabetes appointment non-attendance among self-employed men. The hazard ratio of the attendance promotion intervention versus the control group for the first non-attendance was estimated using a Cox proportional hazard model with robust variance

Among the men, self-employed participants had a higher risk of appointment non-attendance than did full-time employees in Model 1 (adjusted HR, 1.89; 95% CI, 1.18, 3.04) and Model 2 (adjusted HR, 1.84; 95% CI, 1.15, 2.95). The complete case analysis with Model 2 showed similar findings (adjusted HR, 2.07; 95% CI, 1.20, 3.57) (Supplementary Table 2). The appointment attendance promotion intervention significantly reduced the risk of appointment non-attendance among the self-employed participants (HR, 0.51; 95% CI, 0.26, 0.99). The Kaplan–Meier curves separated 80 days after randomization (Fig. 2). The effect of appointment adherence promotion intervention on non-attendance across employment status in men and women is presented in Supplementary Table 3.

The crude incidence rates were lower for the women than for the men, and the estimates had wider confidence intervals (Supplementary Table 1). For women, there was no significant association between employment status and appointment non-attendance in either model (Table 2). These findings were similar to those of the complete case analysis (Supplementary Table 2).

## Discussion

This study analyzed large-scale cluster randomized trial data and showed that self-employed men with type 2 diabetes had a twofold higher risk of non-attendance at regular diabetic outpatient appointments than did those who were full-time employees. Additionally, the subgroup analysis of the J-DOIT2-LT showed that compared with the standard diabetes care, the three-faceted adherence promotion intervention resulted in a lower risk of appointment non-attendance among self-employed men. Our study results suggest that an intervention approach that targets self-employed men (a high-risk target group) is a promising strategy for preventing diabetes progression in the working-age population.

Appointment non-attendance is common for people with diabetes. In one report, 8% of participants missed one-third of their appointments each year [1]. These participants had a mean HbA1c level that was 0.7 points higher than that for participants attending all of their appointments in a year. In addition to poor glycemic control, non-attendance was associated with subsequent hospital admissions or emergency department visits [11, 12]. Several other studies have reported increased cardiovascular complications among non-attenders [13–15]. Among people with type 1 diabetes, appointment non-attendance is also associated with higher all-cause mortality rates [16]. The patient characteristics associated with appointment non-attendance have been diverse and inconsistent between studies. Younger age, smoking, lower socioeconomic status (SES), and higher HbA1c levels have been repeatedly reported as predictors of non-attendance [6]. Several qualitative studies have explored the causes of non-attendance. Illness perception, bureaucratic processes, logistical problems, and healthcare professional–patient relationships have been shown to be important factors affecting appointment attendance [17, 18].

Work-related factors, such as workplace demands, control, support, and relationships, are considered to have an impact on appointment non-attendance among working-age adults [5]. Self-employed individuals have been previously reported to have higher job control and to be more flexible in adjusting their working conditions in the event of chronic illnesses. Among the 1389 participants (50–60 years old) in the English Longitudinal Study of Aging study, self-employed individuals reported significantly higher job autonomy than other employees, at baseline. After the first diagnosis of a chronic illness during the follow-up, self-employed individuals were able to adjust the physical demands of their work, but other employees were less able to do so [19].

Our study findings contrasted with the traditional views of self-employed individuals. In this study, self-employed individuals showed a higher risk of appointment non-attendance than did full-time employees. Patient characteristics, known as predictors of non-attendance, could not fully explain the observed association, in our study. At baseline, HbA1c levels and smoking status were similar between the self-employed and full-time employees. Moreover, the association between self-employment and appointment non-attendance remained, even after adjusting for age, HbA1c level, being treated for diabetes, BMI, and number of previous non-attendance events. These results suggest that the mechanism behind appointment non-attendance among self-employed workers must lay among the work-related factors.

Being self-employed has several disadvantages, such as social isolation, economic insecurity, and inadequate workers’ compensation. For example, in Japan, every employee regardless of full-time or part-time employment is offered up to 20 days of national paid leave, which is not guaranteed among self-employed. Among these, a lack of peers at the workplace may negatively impact health behaviors. A growing body of literature has reported that social interactions affect individual behaviors. In an analysis of the Framingham Heart Study, smoking cessation by a coworker in a small-sized company increased the chances of cessation among the employee’s counterparts by 34% (95% CI, 5, 56) [20]. A study from Austria showed that an individual’s participation in a health-screening program, after moving to a new company, depends on the participation level of their new peers [21]. These findings support the idea that decisions to adhere to health advice are influenced by co-worker behaviors and not solely by the individual. Self-employed participants do not benefit from the collective influence of their peers’ health-related behaviors, possibly leading to the observed increased risk of appointment non-attendance.

Among women, the present analysis did not show a significant association between employment status and appointment non-attendance. The comparisons with full-time employees showed insignificant differences for any of the other employment categories. The absence of any association between appointment attendance and employment classification, among women, may be due to the small number of events and imprecise estimations included in this study. In addition, women generally do more unpaid labor outside of their primary employment; thus, employment status may not sufficiently reflect the barriers to appointment attendance faced by women [5]. Reducing domestic labor or improving health care access from home (e.g., telehealth) would probably be a promising approach toward women.

Several rationales support the suggestion that appointment attendance promotion should be directed toward self-employed individuals. First, the intervention examined in the J-DOIT2-LT was resource-consuming and, thus, requires focused allocation to the high-risk populations. Second, attendance at health checkups is lower among self-employed individuals than among full-time employees [22, 23]. Once self-employed individuals discontinue regular primary care follow-ups, diabetes progression becomes more difficult to identify. Thus, these considerations support interventions that focus on individuals with type 2 diabetes who are at high risk of appointment non-attendance.

Our study had some limitations. First, the study population was limited to participants in a cluster-randomized trial. However, participants were recruited from across the country and most who met the eligibility requirements were enrolled (Fig. 1). Those who met the exclusion criteria in the trial were dependent or required special support to attend their appointments. Thus, the study population was representative of working-age individuals (40–65 years old) with type 2 diabetes who are able to regularly visit their PCPs. Of note, our study results are not directly applicable to the younger individuals (20–39 years old) with type 2 diabetes. Second, several characteristics of the working environment were not collected, such as the company size, employee job class (managerial versus non-managerial), and the types of self-employed jobs involved (e.g., small business owners, independent contractors, farmers). Detailed information will clarify where barriers to appointment attendance exist. Third, educational status information was unavailable. Education predicts both employment status and appointment non-attendance; thus, it may be a confounding factor [24]. We incorporated BMI into Model 2 as a proxy for education. Fourth, we defined appointment non-attendance as a failure to visit a PCP within 2 months of the original appointment. Compared with other studies, we believe that we were more successful at distinguishing clinically relevant appointment non-attendance; however, our exclusion of appointment non-attendance events that did not result in a loss of diabetes care may have been incomplete. Fifth, the number of part-time employees and unemployed was small among men, meaning that the associations with appointment non-attendance in these subgroups could not be addressed with precision.

In conclusion, among adult participants with type 2 diabetes who were followed by PCPs, self-employed men had a higher risk of appointment non-attendance within the one-year follow-up included in this study. Multifaceted appointment adherence promotion interventions for working-age people with type 2 diabetes should target self-employed men.

## Supplementary Information

Below is the link to the electronic supplementary material.

Supplementary file1 (PDF 77 KB)

Supplementary file2 (PDF 93 KB)

## Declarations

### Data and/or Code availability

The data are not publicly available due to them containing information that could compromise research participant privacy/consent.
