# Supplementary material for: Employment status and diabetic outpatient appointment non-attendance in middle to senior working generation with type 2 diabetes: the Japan diabetes outcome intervention trial-2 large‑scale trial 005 (J-DOIT2-LT005)
Source: Acta Diabetol. 2022 Mar 12;59(6):793–801. doi: 10.1007/s00592-022-01869-0 (PMC9085697; doi:10.1007/s00592-022-01869-0)

Supplementary Figure

Izumi Nakayama MD, PhD, Atsushi Goto MD, PhD, Yasuaki Hayashino MD, Hikari Suzuki MD, Katsuya Yamazaki MD, Kazuo Izumi MD, Mitsuhiko Noda MD

Employment status and diabetic outpatient appointment non-attendance in middle to senior working generation with type 2 diabetes: The Japan Diabetes Outcome Intervention Trial-2 Large-scale Trial 005 (J-DOIT2-LT005)

Submitted to Acta Diabetologica

Correspondence to Atushi Goto MD PhD MPH
Department of Health Data Science, Graduate School of Data Science, Yokohama City University,
22-2, Seto, Kanazawa-ku, Yokohama, Kanagawa, 236-0027, Japan
Phone: +8145-787-2311
E-mail: agoto@yokohama-cu.ac.jp

Supplementary figure. Directed acyclic graph illustrating the relationship between employment status and non-attendance.

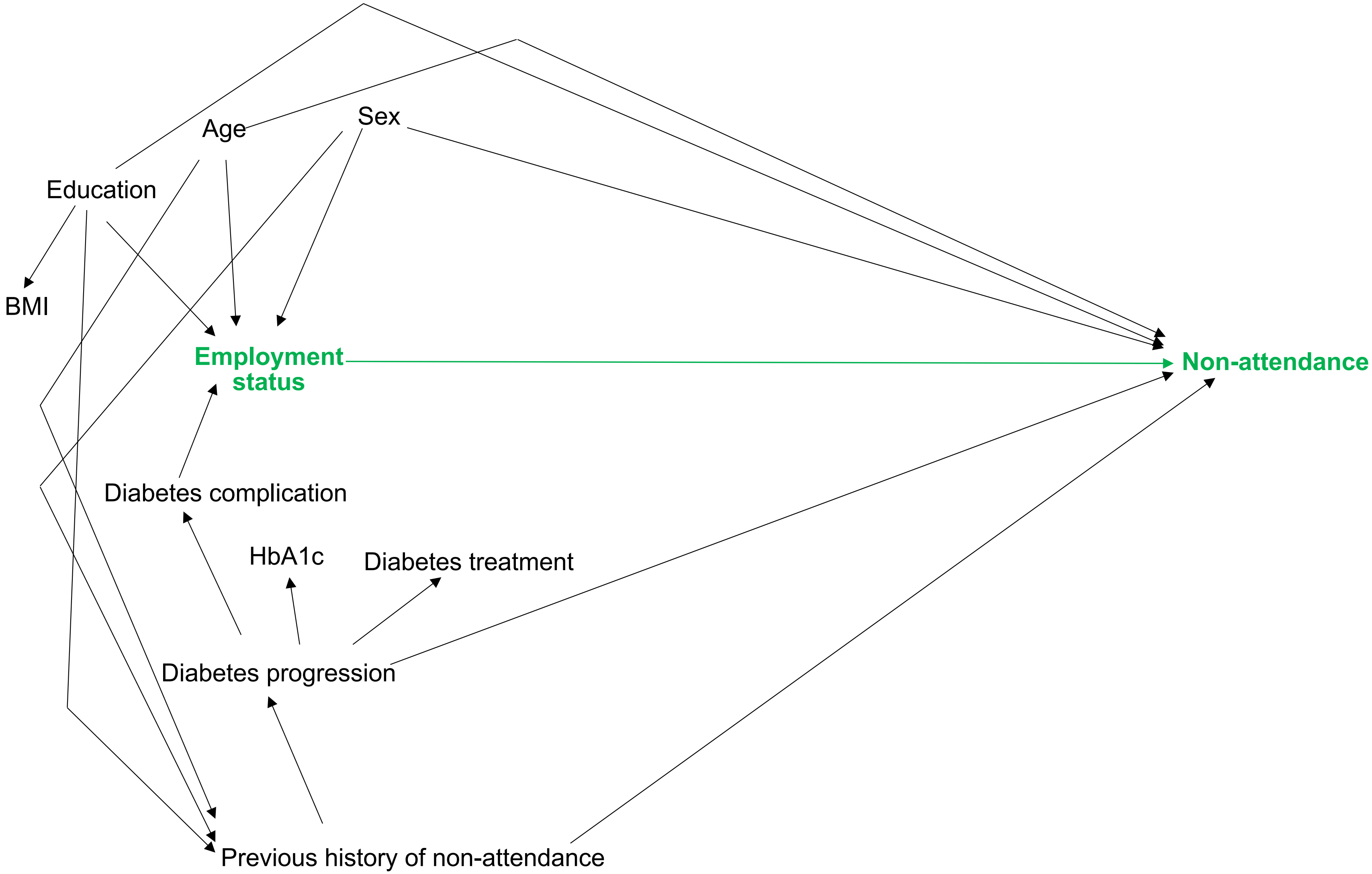

Supplement: Supplementary file 1 — Supplementary file1 (PDF 77 KB) [file 592_2022_1869_MOESM1_ESM.pdf]
