# Supplementary material for: Employment status and diabetic outpatient appointment non-attendance in middle to senior working generation with type 2 diabetes: the Japan diabetes outcome intervention trial-2 large‑scale trial 005 (J-DOIT2-LT005)
Source: Acta Diabetol. 2022 Mar 12;59(6):793–801. doi: 10.1007/s00592-022-01869-0 (PMC9085697; doi:10.1007/s00592-022-01869-0)
Supplement: Supplementary file 2 — Supplementary file2 (PDF 93 KB) [file 592_2022_1869_MOESM2_ESM.pdf]

Supplementary Table

Izumi Nakayama MD, PhD, Atsushi Goto MD, PhD, Yasuaki Hayashino MD, Hikari Suzuki MD, Katsuya Yamazaki MD, Kazuo Izumi MD, Mitsuhiko Noda MD

Employment status and diabetic outpatient appointment non-attendance in middle to senior working generation with type 2 diabetes: The Japan Diabetes Outcome Intervention Trial-2 Large-scale Trial 005 (J-DOIT2-LT005)

Submitted to Acta Diabetologica

Correspondence to Atushi Goto MD PhD MPH
Department of Health Data Science, Graduate School of Data Science, Yokohama City University,
22-2, Seto, Kanazawa-ku, Yokohama, Kanagawa, 236-0027, Japan
Phone: +8145-787-2311
E-mail: agoto@yokohama-cu.ac.jp

Supplementary Table 1. Crude Incidence Rates for Appointment Non-Attendance stratified by Assigned Treatment and Employment Status

Employment status	Person-Years	Non-attendance (N)	Rate/1000 person-years (95% CI)
		Men	
**Control group**			
Full-time	490	41	83.7 (60.0, 113.5)
Self-employed	152	21	138.5 (85.7, 211.7)
Part-time	30	3	99.0 (20.4, 289.2)
Unemployed	56	6	107.1 (85.7, 233.2)
Homemaker	–	–	–
**Intervention group**			
Full-time	354	10	28.2 (13.5, 51.9)
Self-employed	127	9	70.8 (32.4, 134.5)
Part-time	22	0	–
Unemployed	47	0	–
Homemaker	–	–	–
		Women	
**Control group**			
Full-time	87	6	68.9 (25.3, 149.9)
Self-employed	42	2	48.2 (5.8, 174.1)
Part-time	124	13	104.6 (55.7, 178.8)
Unemployed	43	1	23.0 (0.6, 128.4)
Homemaker	132	5	37.8 (12.3, 88.2)
**Intervention group**			
Full-time	60	2	33.6 (4.1, 121.3)
Self-employed	44	0	–
Part-time	113	3	26.5 (5.5, 77.6)
Unemployed	40	2	49.6 (6.0, 179.2)
Homemaker	103	0	–

The crude incidence rates for appointment non-attendance per 1000 person-years and 95% CI were calculated assuming a Poisson distribution. Incidence rates were not calculated for categories without an event. The intervention group received appointment attendance promotion for one year.

Supplementary Table 2. Adjusted Hazard Ratios for Appointment Non-Attendance using Model 1, Model 2 with Complete Cases, and Model 2 with Multiply Imputed Data.

	Hazard ratio (95% CI)		
Employment status	Model 1	Model 2 (complete case analysis)	Model 2 (multiply imputed data)
**Men**			
Full-time	1.0	1.0	1.0
Self-employed	1.89 (1.18, 3.04)	2.07 (1.20, 3.57)	1.84 (1.15, 2.95)
Part-time	1.06 (0.44, 2.53)	0.55 (0.09, 3.36)	1.10 (0.45, 2.66)
Unemployed	1.04 (0.57, 1.88)	1.01 (0.42, 2.42)	1.02 (0.53, 1.93)
Homemaker	–	–	–
**Women**			
Full-time	1.0	1.0	1.0
Self-employed	0.45 (0.13, 1.60)	0.32 (0.03, 3.45)	0.44 (0.10, 1.98)
Part-time	1.27 (0.53, 3.07)	1.48 (0.51, 4.29)	1.29 (0.52, 3.18)
Unemployed	0.70 (0.19, 2.56)	1.16 (0.28, 4.92)	0.81 (0.20, 3.28)
Homemaker	0.41 (0.12, 1.47)	0.29 (0.06, 1.52)	0.51 (0.13, 1.99)

Adjusted hazard ratios for the first appointment non-attendance were determined using a Cox proportional hazard regression model using robust variance. In Model 1, the hazard ratios were adjusted for the baseline age. In Model 2, the hazard ratios were adjusted for age, glycated hemoglobin level, treatment for diabetes, body mass index, and history of previous non-attendance. In the complete case analysis, we used observations without missing values in the model covariates (77.0% [962/1249] of men; 73.3% [558/761] of women). In analyses using multiply imputed data, pooled hazard ratios from 100 copies of the imputed data sets were presented.

Supplementary Table 3. Effect of attendance promotion intervention across employment status.

Men	
	Hazard ratio (95% CI)
Employment status	
Full-time	0.34 (0.16, 0.71)
Self-employed	0.51 (0.27, 0.99)
Part-time	–
Unemployed	–
Homemaker	–
Women	
Full-time	0.48 (0.13, 1.80)
Self-employed	–
Part-time	0.26 (0.04, 1.46)
Unemployed	2.24 (0.23, 21.40)
Homemaker	–

The effect of appointment adherence promotion intervention of the J-DOIT2-LT on non-attendance was evaluated across employment status. Unadjusted hazard ratios for the first appointment non-attendance were determined using a Cox proportional hazard regression model using robust variance. The results were not presented in categories where the regression model did not converge. J-DOIT2-LT, Japan Diabetes Outcome Intervention Trial 2 large-scale trial.
